# Uncovering transcriptomic biomarkers for enhanced diagnosis of methamphetamine use disorder: a comprehensive review

**DOI:** 10.3389/fpsyt.2023.1302994

**Published:** 2024-01-08

**Authors:** Won-Jun Jang, Sooyeun Lee, Chul-Ho Jeong

**Affiliations:** College of Pharmacy, Keimyung University, Daegu, Republic of Korea

**Keywords:** methamphetamine use disorder, non-invasive biomarker, transcriptomic biomarker, diagnosis, addiction

## Abstract

**Introduction:**

Methamphetamine use disorder (MUD) is a chronic relapsing disorder characterized by compulsive Methamphetamine (MA) use despite its detrimental effects on physical, psychological, and social well-being. The development of MUD is a complex process that involves the interplay of genetic, epigenetic, and environmental factors. The treatment of MUD remains a significant challenge, with no FDA-approved pharmacotherapies currently available. Current diagnostic criteria for MUD rely primarily on self-reporting and behavioral assessments, which have inherent limitations owing to their subjective nature. This lack of objective biomarkers and unidimensional approaches may not fully capture the unique features and consequences of MA addiction.

**Methods:**

We performed a literature search for this review using the Boolean search in the PubMed database.

**Results:**

This review explores existing technologies for identifying transcriptomic biomarkers for MUD diagnosis. We examined non-invasive tissues and scrutinized transcriptomic biomarkers relevant to MUD. Additionally, we investigated transcriptomic biomarkers identified for diagnosing, predicting, and monitoring MUD in non-invasive tissues.

**Discussion:**

Developing and validating non-invasive MUD biomarkers could address these limitations, foster more precise and reliable diagnostic approaches, and ultimately enhance the quality of care for individuals with MA addiction.

## 1 Introduction

### 1.1 Methamphetamine and methamphetamine use disorder

Methamphetamine (MA) is a potent synthetic stimulant that disrupts the central nervous system (CNS) and induces physical and psychological dependence (1). MA targets monoamine transporters, particularly dopamine (DA), norepinephrine (NE), and serotonin (5-HT), in the CNS (2). Its primary mechanism of action is the release of neurotransmitters from presynaptic terminals, which predominantly reverses their actions (3). Additionally, MA inhibits monoamine oxidase (MAO), an enzyme responsible for the breakdown of DA, NE, and 5-HT, thereby increasing their availability and enhancing their effects (2). The pharmacological effects of MA include increased arousal, alertness, focus, mood, sociability, and self-esteem (4).

MA use disorder (MUD) is a chronic, relapsing disorder characterized by the compulsive use of MA despite its negative consequences on physical, psychological, and social well-being (5). MUD is a significant global public health problem associated with substantial social and economic costs, including increased mortality and morbidity (6). MUD is associated with a wide range of health issues, including cardiovascular, respiratory, and neurological complications, as well as an increased risk of infections such as HIV and hepatitis C (4, 7, 8). Furthermore, MA use increases the risk of severe clinical withdrawal symptoms, such as depression, anxiety, intense craving for MA, psychosis, and suicidal tendencies (9–11).

The onset of MUD involves a complex interplay between genetic, epigenetic, and environmental factors (12). Furthermore, the causes of MUD may be multifactorial, and there may be correlations with various psychopathological variables, such as emotional disturbances and regulatory disorders in adolescents (13, 14). The neurobiological mechanisms underlying MUD are still not fully understood. However, repeated MA exposure induces neuroadaptations in the brain’s reward system, particularly within the mesolimbic dopamine pathway, including the ventral tegmental area (VTA) and the nucleus accumbens (NAc) (15). These neuroadaptations contribute to the development of compulsive drug-seeking behavior and the persistence of MUD despite its negative consequences (16, 17).

### 1.2 The importance of non-invasive biomarkers for diagnosis and monitoring of MUD

The diagnostic criteria for MUD are primarily based on self-reported questionnaires. In clinical practice, self-report questionnaires are often used to assess substance abuse before and during abstinence (18). Examples of such questionnaires include the Alcohol, Smoking, and Substance Involvement Screening Test (ASSIST) (19), the Drug Abuse Screening Test (DAST) (20), and the Addiction Severity Index (ASI) (21). Although these questionnaires are the most convenient and broadly accessible tools for estimating drug abuse (including MA abuse), there are several limitations in the current diagnostic criteria and methods. First, these questionnaires are inherently subjective and may be influenced by factors such as social desirability bias, memory lapses, and misinterpretation of questions (22). This subjectivity can lead to under-reporting or over-reporting of MA use and related problems, potentially compromising diagnostic accuracy. Questionnaires usually rely on an individual’s honesty and accurate recall of substance use patterns. However, it can sometimes be problematic to evaluate MUD in patients with milder symptoms or in those hesitant to seek treatment (23). Second, the current diagnostic criteria do not consider biological markers of MA exposure or the underlying neurobiological changes associated with MUD. Consequently, they may not accurately capture the full extent of an individual’s MA use or addiction severity. The lack of objective biomarkers also limits the ability to monitor treatment progress and assess the risk of relapse. Research has shown that MA use is associated with distinct neurobiological alterations, psychiatric comorbidities, and functional impairments (1, 24). Therefore, questionnaires must often be combined with quantitative analyses of drugs or metabolites in peripheral biospecimens, such as urine drug tests, as a supplementary tool to confirm recent MA use and strengthen conclusions about drug addiction (25). Consequently, the limitations of the current diagnostic criteria for MUD highlight the need for more accurate and reliable methods of identifying and monitoring individuals with MA addiction.

Non-invasive biomarkers can provide objective and quantifiable measures of MA exposure, offering a more reliable diagnostic approach. They facilitate the early detection of MUD by identifying subtle biological changes that occur before the onset of clinically significant symptoms such as cognitive impairment, psychosis, and neurotoxicity (26). In addition, non-invasive biomarkers can play a crucial role in assessing treatment efficacy as they can provide insight into the underlying biological changes that occur in response to therapeutic interventions (27). By monitoring changes in biomarker levels over time, evaluations can be performed to determine the need for adjustments or alternative approaches. Monitoring the risk of relapse is another important application of non-invasive biomarkers for MUD. Biomarker-level changes can indicate an increased likelihood of relapse, enabling clinicians to implement suitable interventions, such as behavioral therapy or medication, to prevent or attenuate the risk of relapse. Therefore, the real-time monitoring of a patient’s physiological status before and during cravings can help reduce the risk of relapse and improve clinical outcomes (28).

Non-invasive biomarkers have significant potential for improving the diagnosis, early detection, treatment efficacy, and relapse monitoring of MA addiction. Developing these biomarkers requires multidisciplinary research integrating expertise from various fields, including public health, pharmacology, neuroscience, transcriptomics, proteomics, metabolomics, neuroimaging, bioinformatics, and artificial intelligence. As the research progresses, validating these biomarkers in diverse populations and addressing ethical considerations, such as privacy and potential stigmatization, are essential to ensure their successful implementation in clinical practice.

### 1.3 Aim and scope of the review

This review aims to comprehensively analyze existing research and prospects of non-invasive biomarkers for diagnosing MUD. It focuses on the recent developments in identifying and validating biomarkers, particularly in the field of transcriptomics. This review extensively evaluates literature from various scientific disciplines, highlighting relevant and recent findings. It also addresses the limitations and challenges of current diagnostic methods and proposes potential solutions. This review also discusses the importance of interdisciplinary collaboration, biomarker standardization and validation, ethical considerations, and regulatory approval for successful clinical implementation.

## 2 Techniques for identifying transcriptomic MUD biomarkers

Various high-throughput technologies have been employed to comprehensively understand the molecular mechanisms of MUD and identify transcriptomic biomarkers. These technologies provide comprehensive and in-depth information about gene expression profiles, facilitating the detection of transcriptomic changes associated with MUD and assisting researchers in analyzing, diagnosing, and treating the disorder. The 2019 COVID-19 pandemic has catalyzed innovations in diagnostic technologies and bioinformatics (29). RNA and transcriptome analysis technologies have played significant roles in the diagnosis, prognosis prediction, and development of RNA therapeutics (30–32). Here, we discuss the traditional technologies used in RNA and transcriptome analysis and the diagnosis of MUD (Table 1).

**Table 1:** Summary of differences among transcriptome analytic technologies.

Technology		Definition	Concept	Advantages	Disadvantages
qRT-PCR		A technique to quantify relative amounts of RNA molecules	Amplification and detection of mRNA occur simultaneously, allowing monitoring of amplification process over time	High precision, high sensitivity, quantitative measurement of gene expression	Can only analyze a small number of genes, requires rigorous experimental design and sample purification
Microarray		A technique to compare gene expression levels simultaneously	DNA fragments are placed on a fixed array and bind with cDNA from the sample to indicate the expression level of the gene	Can examine thousands of gene expressions at the same time	Cannot provide information on unknown genes, difficulty in detecting low expression genes
mRNA sequencing (mRNA-Seq)		A method using RNA sequencing to determine gene expression levels	Reverse transcription of mRNA to create cDNA, which is sequenced for quantitative analysis of gene expression	High resolution and sensitivity, can explore novel genes or variants	Sequencing process is complex, generates a large amount of data which makes analysis challenging
Non-coding RNA sequencing	microRNA sequencing (miRNA-Seq)	A technique to understand the expression pattern and role of microRNA	Allows the identification and quantification of miRNAs expressed in different cell types and tissues	Can understand the diversity and expression of miRNA	Sequencing is difficult due to short length, and it’s hard to detect low expression miRNAs
	long non-coding RNA sequencing (lncRNA-Seq)	A technique to analyze lncRNA expression and understand their function	lncRNAs play important roles in various biological processes like gene regulation, development, cancer etc.	Can understand the diversity and complexity of lncRNA	Understanding the function and structure of lncRNAs is challenging
Single-cell RNA sequencing (scRNA-Seq)		A method to analyze the transcriptome from individual cells	Helps to understand differences in expression between cells and understand the state of differentiation and function of cells	Can understand differences in expression between cells, understand the state of differentiation and function of cells	RNA extraction and sequencing from single cells is technically difficult, and has a lot of noise

### 2.1 Quantitative real-time PCR

Quantitative real-time PCR (qRT-PCR) is widely used for validating microarray and RNA sequencing (RNA-Seq) experiments. It is a highly sensitive and specialized technique that measures gene expression levels by amplifying target cDNA (33). qRT-PCR was used to validate the expression of several candidate MUD biomarkers identified in transcriptomic studies (34). Although qRT-PCR is a powerful validation tool, it is limited by its low throughput capacity, which makes it unsuitable for large-scale transcriptomic profiling (35). Numerous researchers have recognized the importance of complex interactions and integrated approaches to diseases. As a result, the systems biology and bioinformatics fields have seen significant advancements since the 2000s, propelled by this demand (36, 37). Technologies such as microarray, RNA-seq, non-coding RNA-seq (ncRNA-seq), and single-cell RNA-seq (scRNA-seq) have been developed for high-throughput transcriptome profiling.

### 2.2 Microarray

Microarray technology has been widely employed in transcriptomic studies because of its high-throughput capabilities and ability to analyze thousands of genes simultaneously (38). Microarrays consist of DNA probes immobilized on a solid surface to which complementary RNA targets can hybridize. Following hybridization, the intensity of the fluorescent signal produced by the labeled targets corresponds to the expression levels of the respective genes (39). In the MUD research field, microarray technology is a long-established tool employed as a central analysis component. For instance, this technology has been used to identify differentially expressed genes (DEGs) in response to MA exposure in various animal models and human cell lines (34, 40). However, microarrays have some limitations, including background noise, cross-hybridization, and limited dynamic range (41). Following the COVID-19 pandemic, the commercialization of RNA-seq technology has led to a declining trend in the use of microarray technology.

### 2.3 RNA sequencing

RNA-Seq is a next-generation sequencing (NGS) technique that has revolutionized the study of the transcriptome, offering a comprehensive understanding of the dynamic behavior of biological systems. In contrast to previous methods, such as microarrays, RNA-Seq enables high-throughput analysis of the entire transcriptome, including mRNA and non-coding RNA. This capability allows for the detection of novel transcripts, alternative splicing variants, and mutations/structural variations, providing a comprehensive and accurate picture of gene expression (41). RNA-Seq has transformed disease research by providing insights into gene expression changes associated with various diseases. This allows for the identification of DEGs between normal and diseased states, which can lead to the identification of key disease biomarkers and therapeutic targets. RNA-Seq has also played a crucial role in understanding complex diseases involving multiple genes and pathways, such as cancer, neurodegenerative diseases, and autoimmune disorders (42). Compared to microarray techniques, RNA-Seq has the advantages of high resolution and sensitivity, allowing the detection of low-abundance transcripts and DEGs. It has a wide dynamic range and can identify novel transcripts and splice variants (43, 44). However, mRNA-seq is expensive and generates large amounts of data, thus requiring significant bioinformatics expertise for analysis (45). In the context of MUD, mRNA-Seq has been instrumental in unraveling complex changes in gene expression associated with MA use. In a study by Cadet et al. (34), transcriptional changes in the brains of rats exposed to MA were compared with those in control rats using mRNA-Seq. This study identified numerous DEGs that offer insights into the neurobiological effects of MA (34). Our previous work also investigated gene expression patterns in the rat brain and whisker follicles after MA self-administration using RNA-Seq techniques, including mRNA and miRNA (46–48). Furthermore, RNA-Seq technology has been used to diagnose patients in biomarker discovery studies (49).

### 2.4 Non-coding RNA sequencing

ncRNAs are a class of RNA molecules that do not encode proteins but perform various cellular functions. They can be classified into two main categories based on their length: small non-coding RNAs (sncRNAs <200 nucleotides)—including miRNAs, small interfering RNAs (siRNAs), and piwi-interacting RNAs (piRNAs)—and long ncRNAs (lncRNAs >200 nucleotides). Although sncRNAs are well-known regulators of gene expression at the post-transcriptional level, lncRNAs have been implicated in various biological processes, including transcriptional regulation, chromatin modification, and molecular scaffolding (50).

Small-RNA-Seq is an NGS approach designed to capture and sequence sncRNAs from a sample. This technology has become a powerful tool for discovering and quantifying both known and novel sncRNAs. Given the ability of sncRNAs to regulate gene expression, small RNA-Seqs can provide critical insights into various biological processes and disease states (51). Recent studies have demonstrated that understanding the role of ncRNAs can enhance our understanding of the mechanisms underlying MA-related processes from the perspective of bioinformatics experts. Zhao et al. analyzed miRNAs in the peripheral blood of individuals with MUD to identify differentially expressed miRNAs compared to the control group from the perspective of bioinformatics experts (52). In another study, the response of miRNAs to MA and their effects on the NAc of mice were investigated, revealing their potential contribution to neuronal autophagy, metabolism, and immune responses (53).

### 2.5 Single-cell RNA sequencing

scRNA-Seq is a revolutionary technology that allows profiling of the transcriptome of individual cells. This technique provides a higher resolution of cellular differences and a better understanding of the function of an individual cell in the context of its microenvironment. By capturing and analyzing the transcriptome at the single-cell level, researchers can uncover the diversity of cell types and states within a population that would otherwise be obscured by bulk RNA-Seq measurements (54). scRNA-Seq has proven to be particularly useful in disease research, as it can identify changes in the transcriptome associated with diseases at the individual cell level. This has led to the discovery of novel cell types, the unraveling of disease mechanisms, and the identification of potential therapeutic targets. For example, scRNA-Seq has been used in cancer research to elucidate tumor heterogeneity and the tumor microenvironment, leading to improved therapeutic strategies (55). The key advantage of scRNA-Seq is its ability to dissect cellular heterogeneity and unravel the complexity of biological systems at a resolution that was previously unachievable. It also allows the identification of rare cell types (56). However, scRNA-Seq also has some limitations, including technical noise, the large amount of data generated, the requirement for specialized analytical tools, and the relatively high cost compared to bulk RNA-Seq (57). In 2021, Dang et al. conducted a study investigating the effect of prenatal MA exposure on fetal brain development using a human cerebral organoid model. They observed that MA induces changes in the expression of neuroinflammatory and cytokine gene expression (58).

In conclusion, various technologies that can profile the expression and complex interactions of the transcriptome—including microarrays, mRNA-Seq, ncRNA-Seq, and scRNA-Seq—are required to diagnose, monitor, and treat MUD. qRT-PCR is the most widely used technology for verifying the discovered targets. Each technology has advantages and disadvantages, with RNA-Seq emerging as the preferred method for large-scale transcriptome profiling owing to its high sensitivity and broad dynamic range. Since the 2000s, the importance of non-coding RNA has been highlighted, and research on small RNA has remained popular. As scRNA-Seq technology continues to develop and become cost-effective, it is expected to play an increasingly important role in MUD research, enabling the discovery of cell-specific transcriptomic changes and the derivation of new markers.

## 3 Methods

### 3.1 Literature search

We performed a literature search for this review using the Boolean search in the PubMed database. The search criteria were as follows: (transcript OR transcriptome OR RNA OR mRNA OR miRNA) AND (plasma OR blood OR serum OR urine OR exosome OR hair OR whisker OR saliva OR sweat OR breath OR skin) AND (methamphetamine use disorder OR methamphetamine addiction OR methamphetamine dependence OR methamphetamine abuse OR methamphetamine) NOT cancer.

### 3.2 Inclusion and exclusion criteria

The articles and reviews found through the database search were screened based on the following criteria:

- Studies related to MA use disorders (studies on carcinomas excluded).
- Whole-genome studies to evaluate multiple biomarkers.
- Studies providing performance metrics of biomarkers, such as accuracy or Area Under the Curve (AUC) of the Receiver Operating Characteristic (ROC) curve.
- Studies written in English.
- Studies with the full text available.

**Figure 1:**
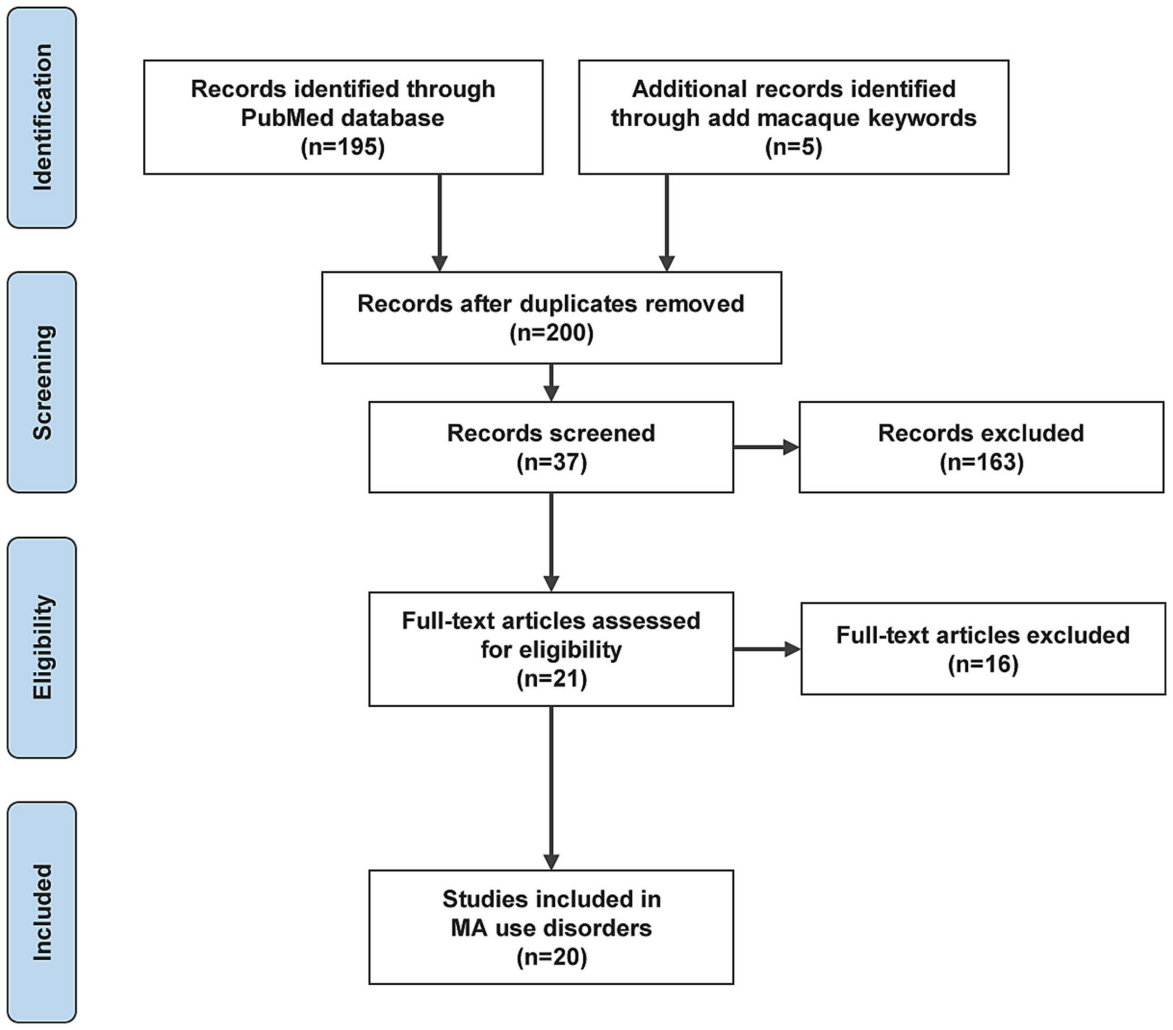
Flow diagram for the literature search and analysis.

The literature review may also include references cited in the retrieved papers. We have represented the process of literature search and analysis with a flow diagram (Figure 1).

## 4 Results

### 4.1 Study selection and overview

**Figure 2:**
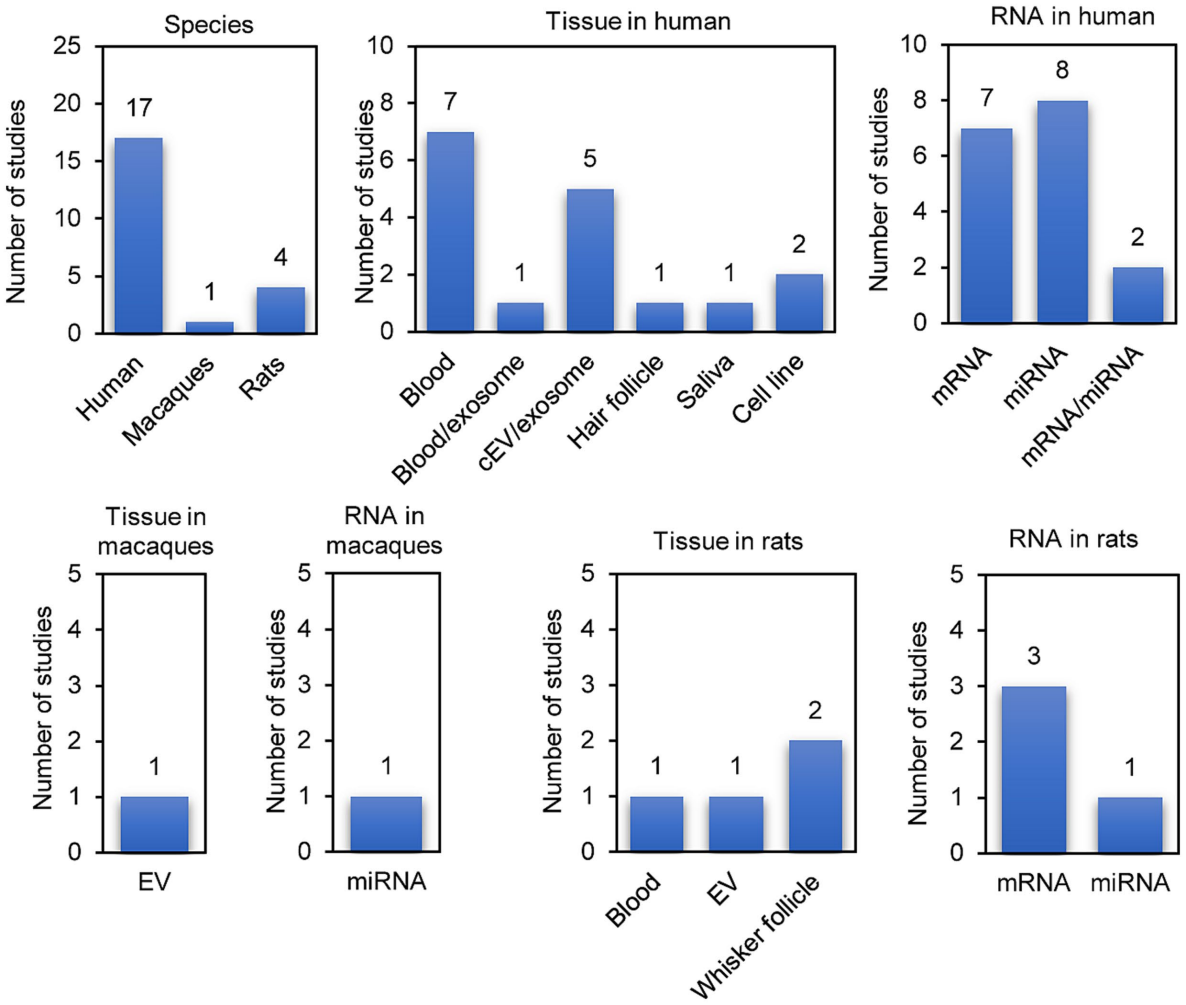
Overview of the transcriptomic biomarker studies identified in the literature search.

Twenty papers that used transcriptomic-based methods to identify biomarkers of MUD or MA exposure were identified (Table 2). These 20 studies were designed to identify biomarkers by comparing MUD versus non-MUD, MA users versus non-users, MUD versus MUD treatment, or similar populations (Figure 2). Seventeen studies investigated biomarkers of MUD in humans, one in macaques and three in rats. Among the human studies, seven used blood tissue, five used circulating extracellular vesicles (cEVs)/exosomes, one used blood/exosomes, one used saliva, two used cell lines, and one used hair follicles for their investigations. Additionally, seven studies focused on mRNA biomarkers, eight on miRNA biomarkers, and two on both mRNA and miRNAs simultaneously. Many studies have evaluated the effects of MA on the transcriptomes of peripheral tissues without providing performance measures for biomarkers. Of the 20 studies, only seven included performance measures of the biomarkers. These seven studies were conducted on humans. Three studies used blood, two used cEV/exosomes, one used blood/exosome, and the other used hair follicles. Five studies measured miRNAs, and two study measured mRNA levels. Overall, the peripheral transcriptome signatures could distinguish between MUD and non-MUD (or similar MA-related phenotypes, depending on the study).

**Table 2:** MUD transcriptome studies in non-invasive tissues.

References	Species	Sources	RNA type	Methodology	Condition	Biomarkers	Biomarker performance
Breen et al. (59)	Human	Blood	mRNA	RNA-Seq	• MA dependence with MAP (*n* = 10) • MA dependence without MAP (*n* = 10) • Healthy control (*n* = 10)	ELK3, MAGEE1, RNF138P1, TBC1D2, DDRGK1, MTHFSD, ARL6, FAM169A, ZSCAN5A, FBN1, ZNF821, FBP1, C7orf11, PHLDB2	BRB-array tools-supervised classification methods using RFE and LOOCV
Zhang et al. (60)	Human	Cell line	mRNA miRNA	qRT-PCR	• MA (*n* = 124) • Healthy control (*n* = 57)	GRIA2, miR-181a	
Niu et al. (61)	Human	Blood	mRNA	Microarray	• METH-dependent participants – TPM (*n* = 69) – placebo (*n* = 71)	PML, SASH1, FPR1, GABARAPL1, CSNK1A1, CTNND1, CXCR4, DTX1, MAPK14, PLEKHF2, PSMB2, PSMD1, PTEN	
Gu et al. (62)	Human	Blood	miRNA	Microarray	• MA abuser (*n* = 42) • Control (*n* = 42)	miR-9-3p	ROC-AUC
Wei et al. (63)	Human	Blood	mRNA	qRT-PCR	• Healthy control subjects (*n* = 113) • Methamphetamine-dependent patients (*n* = 118)	5-HT1A, 5-HT1B, Dopamine-D1, and Dopamine-D2 receptors	
Yang et al. (64)	Human	Blood	mRNA	qRT-PCR	• METH addicts without depression (*n* = 25) • METH addicts with depression (*n* = 47)	TrkB	
Rezai Moradali et al. (65)	Human	Blood	miRNA	qRT-PCR	• MA (*n* = 60) • Healthy control (*n* = 60)	miR-127, miR-132	ROC-AUC
Zhao et al. (52)	Human	Blood	miRNA	Microarray	• MA-dependent patients (*n* = 4) • Normal controls (*n* = 4)	miR-181a, miR-15b, miR-let-7e, miR-let-7d	
Xu et al. (66)	Human	Blood & exosome	miRNA	Microarray, miRNA-Seq, qRT-PCR	• MA patients (*n* = 82) • Healthy control (*n* = 50)	miR-320	ROC-AUC
Kim et al. (67)	Human	cEVs	miRNA	qRT-PCR	• MA abstinence (*n* = 37) • Healthy control (*n* = 35)	miR-137	ROC-AUC
Burns and Ciborowski (68)	Human	Cell line	mRNA	qRT-PCR	• Control • Meth (2 h) • Meth (6 h)	TNF, CXCL1, IL-8, CCL7	
Sandau et al. (69)	Human	cEVs	miRNA	miRNA array	• MA-ACT (*n* = 10) • CTL (*n* = 10)	miR-301a-3p, miR-382-5p, miR-628-5p	
Chen et al. (70)	Human	Exosome	miRNA	small RNA-Seq	• MA dependence – withdraw 7 days (*n* = 20) – 3 month (*n* = 20) – 12 month (*n* = 20) • Healthy control (*n* = 20)	miR-744-5p	ROC-AUC
Wu et al. (71)	Human	Exosome	mRNA, miRNA, lncRNA	RNA-Seq	• MUD patients, AW (*n* = 22) • MUD patients, PW (*n* = 29) • Healthy control (*n* = 31)	IL-1b, IL-9, IL-15, Basic FGF, and MIP1a, IL-1ra, IL-6, Eotaxin IP-10, VEGF, RANTES, IL-7, IL-12p70	
Jang et al. (49)	Human	Hair follicle	mRNA	RNA-Seq	• Control (*n* = 29) • Methamphetamine (*n* = 23) • Recovery (*n* = 11)	PSMA2, RAC3, PPP1R12A, DVL1, SUFU, APC2, KLC3, NDUFA4, FADD, APOE	ROC-AUC
Nohesara et al. (72)	Human	Saliva	mRNA	qRT-PCR	• MA without psychotic experience (*n* = 25) • MA with psychotic experience (*n* = 25) • Normal control (*n* = 25)	DRD3, DRD4, MB-COMT, AKT1	
Chand et al. (73)	• Human • Macaque • Rat	sEVs/exomeres	miRNA	qRT-PCR	• Rhesus macaque: Control (*n* = 5) / MA (*n* = 5) • Rat: Control (*n* = 14) / MA (*n* = 12) • Human: Control (*n* = 10) / MA (*n* = 10)	miRNA-29a	
Tavakkolifard et al. (74)	Rat	Blood	mRNA	qRT-PCR	• MA non-prefer (*n* = 21) • MA prefer (*n* = 11)	OX1R	
Song et al. (46)	Rat	Whisker follicle	mRNA	RNA-Seq	• CON: before self-administration (*n* = 6) • MASA: MA self-administration (*n* = 6) • WD: MA self-administration after withdrawal (*n* = 6)	Vcl, Nrp1, Itgb1, Tgfb1, Src, Ywhab, Nfkb2, Rela, Sdc2, Akt1, App, Mapk14, Egfr	
Jang et al. (47)	Rat	Whisker follicle	mRNA	RNA-Seq	• MA self-administration (*n* = 6) • SA(saline) self-administration (*n* = 6)	App, Per1, Ddit4, Tagln	

cEVs, circulating Extracellular vesicle; miRNA, microRNA; lncRNA, long non-coding RNA; MA, Methamphetamine; MAP, MA-associated psychosis; TPM, Topiramate; METH, Methamphetamine; MUD, methamphetamine use disorder; RFE, Recursive feature elimination; LOOCV, Leave-one-out cross-validation; ROC, Receiver operating characteristic curve; AUC, Area Under the Curve.

Thirteen studies did not measure biomarkers. These studies did not meet the inclusion criteria but formed the basis of RNA biomarkers for MUD; hence, they were separated. Nine studies were conducted in humans, and three were performed in experimental animals, targeting rats. Additionally, one study was conducted involving macaques, rats, and humans simultaneously. Four studies used whole blood, two used cell lines, one used saliva, and three used plasma EVs. Three studies measured miRNAs, two simultaneously measured miRNAs and mRNA, and five measured mRNA.

In several studies, there have been reports suggesting that gender differences are an essential consideration not only in the transcriptomic biomarker research for MUD but also for substance use disorder (SUD). Differences in transcriptomic expression based on gender can impact the accuracy of diagnostic biomarkers, potentially leading to a decrease in the efficacy of diagnosis and treatment. Out of the 20 studies we reviewed, 4 discussed gender differences (63, 66, 69, 72). Two of these studies demonstrated that the effects of MA are more pronounced in females and have empirically confirmed significant differences in elements like miRNA and DA through direct experimentation (63, 66). Additionally, two other studies mentioned the heightened sensitivity to MA effects in females (69, 72). The remaining 16 studies either used participants of the same gender, utilized animals, or included both genders but did not consider gender differences in their discussions.

### 4.2 Non-invasive MUD biomarkers

The identification of biomarkers for MUD in animals and humans is primarily conducted using brain tissue. However, for the diagnosis and monitoring of clinical treatments, non-invasive biomarkers in accessible tissues are necessary. The identification of non-invasive transcriptomic biomarkers from biological fluids and hair has gained significant attention, as these are more easily accessible and less invasive than other sampling methods (75). Here, we discuss the transcriptomic biomarkers identified to date for the diagnosis, prediction, and treatment monitoring of MUD in non-invasive tissues. Unless otherwise specified, we focus our discussion solely on MA-related content in research papers involving several drugs.

#### 4.2.1 Biomarkers in blood

Blood, specifically peripheral blood, is one of the most common sources for transcriptome research on various diseases (including juvenile arthritis, hypertension, cancer, chronic fatigue, neuronal injuries, and metabolic disorders) because of its relatively non-invasive collection procedure (76, 77). Blood samples can be collected relatively easily and offer the advantage of being readily applicable to various analytical methods and techniques, such as microarray and qRT-PCR. However, the major disadvantage of blood samples is the high background noise owing to the different cell types and dynamic changes in blood components in response to various external stimuli (77). Blood biomarkers for MUD have been investigated in several studies, and some candidates have shown promise as potential diagnostic markers. Among the 19 studies investigated, 8 reported transcriptome and transcriptome profiling studies using samples derived from blood or peripheral blood. Among these studies, four measured the biomarker performance. Additionally, seven studies focused on human participants, whereas one study was conducted using rat subjects.

Wei et al. investigated neurotransmitter levels and associated receptors in the bloodstream of MA-addicted individuals compared with healthy controls (*n* = 118 and *n* = 113, respectively) using ELISA and qRT-PCR (63). The study revealed significantly decreased blood 5-HT levels and increased blood DA and glutamate levels in MA-dependent patients but no significant difference in NE levels. Surprisingly, no significant correlation was found between the mRNA expression of neurotransmitter receptors and serum neurotransmitter concentration. Despite the changes in the blood concentrations of neurotransmitters, there were no significant changes in the expression of the corresponding receptor mRNA. Additionally, there was no correlation between the blood concentrations of neurotransmitters and the expression of related receptor mRNA.

Breen et al. performed RNA-Seq blood transcriptome profiling of subjects with MA-associated psychosis (MAP; *n* = 10), MA individuals without psychosis (MA; *n* = 10), and healthy controls (*n* = 10) to identify potential blood biomarkers of MAP (59). Employing a weighted gene co-expression network analysis (WGCNA), they identified 25 candidate biomarkers for subjects with MA and 20 for MAP. These biomarkers included 14 genes associated with the circadian clock, genetic transcription, RNA degradation, and ubiquitin-mediated proteolysis. These potential biomarkers could distinguish MA patients from healthy controls and provide insights into the biological mechanisms underlying psychosis.

In a study of mental illness, Yang et al. assessed neurotrophins and their receptors in peripheral blood mononuclear cells related to the antidepressant effects of exercise therapy during long-term MA abstinence (64). They targeted male MA addicts with or without depression and divided them into control and exercise groups. Plasma brain-derived neurotrophic factor (BDNF), neurotrophin-3 (NT-3), neurotrophin-4 (NT-4), nerve growth factor (NGF), and pro-BDNF levels were measured using ELISA. Neurotrophin receptor expressions [tropomyosin receptor kinase A (TrkA), tropomyosin receptor kinase B-full length (TrkB-FL), tropomyosin receptor kinase B-truncated (TrkB-T1), tropomyosin receptor kinase C (TrkC), and low-affinity neurotrophin receptor (P75NTR)] in peripheral blood mononuclear cells were detected through qRT-PCR. The study found a significant association between plasma NT-4 levels and depression and a remarkable decrease in TrkB-FL and TrkB-T1 mRNA expression in peripheral blood mononuclear cells after exercise. Exercise appears to have an antidepressant effect in patients recovering from MA addiction, indicating its potential as a beneficial treatment for these patients.

Niu et al. conducted a study to identify biomarkers for tracking changes in gene expression during therapeutic interventions for MA dependence using topiramate (TPM) (61). They analyzed microarray gene expression data from 8- and 12-week TPM responders and identified 1,381 genes in the 8-week group, with 359 genes common to both time points and 300 genes unique to TPM responders. Ingenuity pathway analysis revealed the presence of the phosphoinositide 3-kinase/AKT serine/threonine kinase 1 (AKT1) signaling pathway in both TPM groups. Certain genes were consistently downregulated, including glycogen synthase 1 (GYS1), heat shock protein 90 beta family member 1 (HSP90B1), NFKB inhibitor epsilon (NFKBIE), protein phosphatase 2 regulatory subunit B’delta (PPP2R5D), RAS related (RRAS), and tumor protein p53 (TP53). In contrast, phosphatase and tensin homolog (PTEN) were consistently upregulated. Different molecular interaction networks were detected in the 8- and 12-week TPM groups, suggesting a combined effect of TPM and MA on multiple molecular pathways, leading to the attenuation of MA withdrawal symptoms. This study identified enriched pathways related to neuronal function, synaptic plasticity, signal transduction, inflammation, immune function, and oxidative stress response, offering potential insights for more effective treatments for MA dependence.

In another study related to MA dependence, Tavakkolifard et al. investigated the correlation between the gene expression levels of the orexin-1 receptor (OX1R) in the rat prefrontal cortex (PFC) and blood lymphocytes and susceptibility to MA dependence and novelty-seeking behavior (74). This study was conducted in male Wistar rats to evaluate OX1R expression using real-time PCR. Novelty-seeking behavior was assessed using the novel object recognition test, and susceptibility to MA abuse was examined using voluntary MA oral consumption tests. The results showed that rats in the MA-preferring group had significantly higher OX1R expression in both blood lymphocytes and the PFC than rats in the non-preferring group. This study suggested that MA abuse may impact orexin regulation. Moreover, upregulation of OX1R mRNA expression in lymphocytes and the PFC could predict vulnerability to MA consumption and novelty-seeking behavior. This finding may help predict sensitivity to the reward effects of MA and identify individuals with higher novelty-seeking tendencies.

Small RNAs, particularly miRNAs, have gained attention for their roles in gene regulation since the early 2000s (78). miRNAs are small non-coding RNA molecules that post-transcriptionally regulate gene expression by binding to target mRNA 3′ untranslated regions (79). The dysregulation of miRNAs can contribute to various diseases, including cancer, cardiovascular diseases, and neurological disorders (80, 81). Research on miRNAs as non-invasive biomarkers for diseases has been growing because of their stability in bodily fluids and tissues and their potential as non-invasive markers for diagnosis and prognosis. Studies have explored the effect of MA on miRNA expression profiles in the context of MA-induced neurotoxicity; however, more comprehensive research is required.

Zhao et al. investigated plasma miRNA expression in patients with MUD (52). They found six downregulated miRNAs (miR-181a, miR-15b, miR-let-7e, and miR-let-7d) in patients with MUDs compared with healthy controls. These altered miRNAs were negatively correlated with the frequency of drug use. They and others also revealed that miR-181a could bind to the mRNA transcripts of the human glutamate receptor genes, glutamate ionotropic receptor AMPA type subunit 2 (GRIA2), and gamma-aminobutyric acid receptor subunit alpha-1 (GABRA1) (60). Validation experiments confirmed elevated GRIA2 expression in patients with MUD, and cell-based studies supported miR-181a’s role in regulating GRIA2 expression. This study highlights the significant roles of miR-181a and GRIA2 in MUD.

Gu et al. explored circulating miRNA expression in drug addicts as a non-invasive diagnostic tool for drug abuse (62). Microarray analysis identified 109 significantly altered miRNAs in MA addicts, with miR-9-3p exhibiting increased expression. This suggests the potential of altered serum miRNAs as adjunct tools for identifying individuals with drug abuse or addiction.

Moradaali et al. investigated the expression levels of miR-127 and miR-132 in MA abusers in Iran (65). They found significantly higher expression of miRNA-127 and miRNA-132 in MA abusers than in healthy controls, indicating their possible involvement in the pathophysiology of MA abuse.

#### 4.2.2 Biomarkers in cEVs, exosome

Circulating extracellular vesicles (cEVs) have gained recognition as crucial tools for intercellular communication in biomedical research (82, 83). They encompass various entities such as ectosomes, microparticles, microvesicles, exosomes, and oncosomes, which are released not only during cellular death but also during the normal functioning of healthy cells. These vesicles found in the circulatory system encapsulate biomolecules (RNA, proteins, and metabolites) and hold promise as disease biomarkers, influencing physiological functions (83).

Exosomes are small extracellular vesicles (30–150 nm) that play a vital role in cell-to-cell communication and addiction; they contain biomolecules such as proteins, lipids, mRNA, miRNA, and lncRNA. They are secreted by various cell types and are found in diverse biofluids such as blood, urine, and cerebrospinal fluid (84–86). Owing to their easy isolation and detection from body fluids, exosomes and cEVs offer the potential for disease etiology elucidation, early diagnosis, tracking, and diagnostic marker development (87, 88). However, determining their origin and reflecting specific biological states remains challenging (89).

Recent studies have highlighted exosomes as a potential source of non-invasive biomarkers for MUD (67). One study focused on miRNA markers for MA withdrawal symptoms and found that cEV miR-137 was significantly reduced in patients with MA withdrawal symptoms compared with healthy controls. cEV miR-137 showed consistent diagnostic power regardless of the duration of MA withdrawal symptoms or period of MA use. Interestingly, cEV miR-137 interacts with age, demonstrating different diagnostic power for distinguishing MA withdrawal symptoms more effectively in younger populations. Factors such as the duration of MA use or withdrawal symptoms, smoking status, depression, and antidepressant treatment did not affect the decrease in cEV miR-137 levels caused by MA withdrawal symptoms. These findings suggest that cEV miR-137 has the potential to serve as a stable and accurate diagnostic marker for MA withdrawal.

Sandau et al. investigated the effect of MA on human plasma extracellular vesicles and their miRNA cargo (69). These findings reveal that MA use influences extracellular vesicles and their miRNA content, highlighting the importance of further research to explore their roles in addiction, recovery, and relapse mechanisms. This study is the first to analyze plasma extracellular vesicles and their miRNA cargo in both MA users and controls. Notably, the MA use group exhibited an increase in the tetraspanin markers (CD9, CD63, and CD81) of extracellular vesicles. In contrast, there was no such increase in coagulation, platelets, and red blood cell-derived extracellular vesicles. Moreover, among the 169 plasma EV-miRNAs, eight of them (miR-223-5p, miR-301a-3p, miR-32-5p, miR-191-5p, miR-142-5p, miR-29a-3p, miR-199a-3p, and miR-579-3p) exhibited noteworthy characteristics in the MA use group based on multiple statistical criteria.

Interestingly, 15 miRNAs of interest were identified in smokers, two of which overlapped with those in the MA-use group. Three miRNAs (miR-301a-3p, miR-382-5p, and miR-628) in the MA use group were significantly associated with clinical features of MA use. Their target predictions were linked to pathways related to MA use, particularly cardiovascular disease and neuroinflammation. These findings emphasize the impact of MA use on extracellular vesicles and their miRNA content, underscoring the need for further research to investigate their role in addiction, recovery, and relapse mechanisms. Additionally, this study aimed to explore the potential of plasma extracellular vesicles as valuable clinical biomarkers for monitoring recovery from MA use disorder. Furthermore, to gain insights into the pathways regulated by cEV and their molecular cargo, mechanistic studies investigating the subtypes of extracellular vesicles (neuron-derived vs. microglia-derived extracellular vesicles) and their targets are necessary.

A study conducted by Wu et al. focused on investigating the severe damage caused by MA addiction and withdrawal from the immune and neural systems (71). However, much of its etiology remains unknown. The researchers examined the peripheral cytokine and exosomal transcriptome regulatory networks in patients with MUD. Among the 51 participants, 22 were in the acute withdrawal (AW) phase, 29 were in the prolonged withdrawal (PW) phase, and 31 were age-and sex-matched healthy controls (HCs). Compared to HCs, the levels of Interleukin (IL)-1β, IL-9, IL-15, Basic FGF, and MIP1a significantly decreased. In contrast, IL-1rα, IL-6, Eotaxin IP-10, vascular endothelial growth factor (VEGF), and regulated up activation, normal T cell expressed and secreted (RANTES) levels increased during the AW phase. Most disturbances were fully or partially restored to baseline levels during the PW phase. However, the levels of cytokines, such as IL-6, IL-7, and IL-12p70, continued to increase even after 1 year of withdrawal. Additionally, the counts of CD3+ and CD4 + T cells significantly decreased in the AW phase; this reduction was restored to baseline during the PW phase.

Conversely, no statistically significant changes were observed in CD8 + T, NK, and B cells. Furthermore, researchers have profiled exosomal mRNA and lncRNAs and established an lncRNA-miRNA-mRNA network associated with the AW and PW phases. Notably, chemical marker signaling markedly increased during the AW phase, whereas differentially expressed mRNAs/lincRNAs were significantly enriched in neurodegenerative diseases during the PW phase. In conclusion, this study identified a series of cytokines and exosomal mRNA/lncRNA regulatory networks linked to MA withdrawal. The study provides an experimental and theoretical foundation that could be valuable for further understanding the etiology of withdrawal symptoms in MUD.

Xu et al. aimed to identify miRNA biomarkers in the blood plasma and exosomes of patients through multi-omics research (66). This study included 82 MA patients and 50 healthy controls. Plasma miRNA analyses used samples from five patients with MA and five healthy controls, whereas miRNA analyses of exosomes used samples from 39 patients with MA and 21 healthy controls. They screened 2006 miRNAs in plasma using microarray technology and 758 miRNAs in exosomes using miRNA-Seq technology. Of these, 603 miRNAs exhibited common expression changes in both the plasma and exosomes. Among them, miRNAs satisfying the conditions of *p* < 0.01 and fold-change>2.0 were ultimately selected. Interestingly, two miRNAs (miR-320a-3p and miR-320c) were identified in the plasma, and five miRNAs (miR-320a-3p, miR-320b-1, miR-320b-2, miR-320c-1, and miR-320c-2) were identified in the exosomes. The miR-320 family in both plasma and exosomes was validated using qRT-PCR and showed significantly increased expression in patients with MA compared to healthy controls. Diagnostic power was assessed using the Area Under the Curve (AUC) of the Receiver Operating Characteristic (ROC) curve, with AUC values of 0.751 and 0.962 for miR-320 in plasma and exosomes, respectively. Increased plasma miR-320 levels were positively associated with smoking, age at onset, and daily use of MA. Target pathway predictions related to miR-320 included cardiovascular diseases, synaptic plasticity, and neuroinflammation. In conclusion, this study highlights miR-320 in the plasma and exosomes as a potential blood-based biomarker for diagnosing MUD.

Chen et al. characterized changes in neurotransmitter and exosomal miRNA profiles during heroin and MA withdrawal (70). This study also sought to determine their associations with psychiatric comorbidities in a large group of patients with substance use disorder (SUD). A list of DEGs, including the presenilin enhancer gamma-secretase subunit (PSENEN), ferredoxin 2 (FDX1L), VPS37D subunit of ESCRT-I (VPS37D), spectrin beta non-erythrocytic 4 (SPTBN4), serine/threonine-protein phosphatase 5 (PPP5C), and family with sequence similarity of 57 member B (FAM57B) were identified as potential direct or indirect targets of hsa-miR-744–5p. The dysregulated miRNA signatures, including hsa-miR-451a and hsa-miR-21a, resulted in an AUC of 0.966 and 0.861 for predicting SUD, respectively. The identified DEGs were mainly involved in neurodegenerative diseases rather than psychiatric disorders. For example, PSENEN, which is associated with late-onset Alzheimer’s disease, is a key regulator of the gamma-secretase complex that is involved in amyloid beta 42 peptide production. SPTBN4 disorders are characterized by severe developmental delays or intellectual disability. The study suggests that the miRNA content of circulating exosomes represents a biomolecular “fingerprint” of substance withdrawal progression, potentially contributing to psychiatric symptoms. This study is significant in the field of substance withdrawal, which is lacking in molecular biomarker and related mechanistic studies.

Burns et al. aimed to identify a miRNA marker of MA abstinence in cEVs (68). Researchers quantified miR-137 in the cEVs of patients with MA abstinence and compared them to those of healthy controls. The study included 37 patients with MA abstinence and 35 age-matched healthy controls diagnosed with SUD for MA, according to the Diagnostic and Statistical Manual of Mental Disorders, Fifth Edition (DSM-5). Blood samples were collected from all the patients. This study found that the reduction in cEV miR-137 was stable irrespective of MA use or abstinence duration. Interestingly, an interaction was observed with age; control participants displayed an aging-dependent reduction in cEV miR-137, whereas MA-abstinent patients showed an age-dependent increase in cEV miR-137. This study demonstrates that miR-137 in cEVs holds high potential as a stable and accurate diagnostic marker for MA abstinence syndrome.

Chand et al. demonstrated evidence that miR-29a is elevated in brain-derived EVs (BDE) and in EVs extracted from the blood using chronic methamphetamine (MA) exposure models in non-human primates (macaques) and rodents (rats) (73). Furthermore, the researchers discovered that miR-29a is abundantly expressed in EV pools composed of small EVs and exomers. They showed that miR-29a plays a crucial role in MA-induced inflammation and synaptodendritic damage. By extracting EVs from the blood of individuals diagnosed with MUD, the researchers provide evidence suggesting that miR-29a could serve as a biomarker for detecting neural damage in individuals diagnosed with MUD.

#### 4.2.3 Biomarkers in saliva

Saliva is a complex biofluid secreted from the major and minor salivary glands and serves as an important source of biomarkers reflecting the body’s internal state (90, 91). It contains proteins, transcripts, microbes, cells, hormones, and antibodies. Saliva offers clear advantages for biomarker research owing to its non-invasive collection, allowing frequent sampling without special treatment or preservation (92). Additionally, saliva contains various forms of RNA, enabling the assessment of the cell transcriptome status. However, its relatively low RNA concentration may limit its sensitivity and specificity during analysis, which demands high accuracy (93). The complexity of the diverse components of saliva can complicate the process of isolating or purifying specific components (94). Despite these challenges, transcriptome analysis of saliva can be used for early disease diagnosis and monitoring, providing insights into oral cancer, metabolic syndrome, autoimmune diseases, and potential diagnostic markers (95). As a non-invasive biological fluid, saliva holds promise as a valuable source of MUD biomarkers.

Nohesara et al. investigated the epigenetic and expression changes induced by MA in key genes associated with psychosis (72). The study included patients with MA dependence, with and without psychosis, along with control subjects; each group consisted of 25 individuals. RNA and DNA were extracted from the saliva samples of patients with MA dependence and psychosis, MA dependence without psychosis, and control subjects. The study found significant DNA hypomethylation of the promoter regions of the dopamine receptor D3 (DRD3), dopamine receptor D4 (DRD4), membrane-bound catechol-O-methyltransferase (MB-COMT), and AKT1 genes associated with increased expression of the corresponding genes in patients with MA psychosis. This was observed to a lesser degree in some candidate genes in non-psychotic patients than in control subjects.

These genes are related to dopamine receptors (DRD1, DRD2, DRD3, and DRD4), which play roles in reward, motivation, memory, and other functions, potentially impacting psychiatric disorders such as psychosis. MB-COMT is involved in dopamine breakdown, and changes in its expression can influence dopamine levels and behavior. Glutamate Decarboxylase 1 (GAD1) affects the neurotransmitter gamma-aminobutyric acid (GABA) levels, potentially influencing mood and cognition. AKT1, which is associated with cell survival and metabolic pathways, has implications for psychiatric disorders such as schizophrenia and psychosis. This study provides evidence that MA dependence is associated with reduced DNA methylation and increased expression of key genes involved in the pathogenesis of psychotic disorders.

#### 4.2.4 Biomarkers in hair roots: hair follicles and whisker follicles

Hair samples are desirable sources for SUD diagnosis because of their non-invasive collection, long detection window, and resistance to external environmental conditions (96, 97). Addictive drugs and their metabolites are incorporated into the hair matrix through blood circulation, sweat, and sebum, resulting in their accumulation in the hair shaft (98). Given that hair grows at a rate of approximately 1 cm per month, drug use can be retrospectively assessed by analyzing segments of the hair shaft (99, 100). This characteristic allows the detection of drug use over several months or even years, depending on hair length (101). Consequently, hair analysis can effectively track the drug usage history of heavy drug users (99, 102). Moreover, hair analysis is less invasive and more tamper-resistant than other methods, such as blood and urine testing. It also avoids common challenges faced in urine testing, such as adulteration or substitution (103). Furthermore, hair analysis has a higher detection limit for various drugs than blood or saliva samples, making analysis easier and more accurate (104).

Research has shown that melanin, the primary pigment in hair, can bind to drugs, leading to higher drug concentrations in darker hair. This variability can result in differences in drug detection levels among individuals with different hair characteristics (100). To minimize these variations, some researchers have suggested using standardized hair sampling procedures and population-specific cut-off values (103). Recent studies have focused on profiling hair and hair follicles’ endogenous metabolome to identify MA use biomarkers (105, 106).

Despite the numerous advantages of hair analysis, certain limitations must be considered when detecting addictive drugs and their metabolites. One challenge is the potential for external contamination, as hair can come into contact with drugs through environmental exposure, such as the passive inhalation of drug particles. To address this issue, various decontamination procedures, including the use of organic solvents and detergents, have been developed to remove external contamination (103). Another limitation is the detection window for hair analysis, which is influenced by factors such as hair growth rate, cosmetic treatments, and environmental conditions, which may impact the interpretation of the results (100, 101, 107).

Maekawa et al. proposed using scalp follicles to identify genes related to brain diseases because the brain and scalp follicles share a developmental origin as ectoderm-derived tissues (108). By examining gene expression in hair follicles and postmortem brain tissue samples from patients with autism, they proposed hair follicles as an alternative tool to reflect the disease state of the central nervous system, including conditions such as autism and chronic psychosis (108). Given that MUD is classified as a brain disease, discovering MUD biomarkers through transcriptome profiling of hair follicles is a valid approach. Our research group is currently conducting studies on transcriptome profiling and biomarker discovery related to MA use in rat whisker follicles and human hair follicles, which will be presented here.

Our group has studied the gene expression profile of MA-induced reward effects in a rat model of MA self-administration (46). Using RNA-seq, we investigated changes in gene expression in rat whisker follicles before self-administration, after MA self-administration, and after withdrawal. We identified six statistically significant gene expression patterns and constructed a functional network of 43 core genes, including HSP90-beta 1 (HSP90AB1), RAC-alpha serine/threonine-protein kinase (AKT1), and proto-oncogene non-receptor tyrosine kinase (SRC). These genes are associated with drug addiction, suggesting their importance in MA addiction. Notably, HSP90AB1 shows increased expression in the rat frontal cortex after morphine self-administration (109), whereas AKT1 and SRC are linked to excessive alcohol consumption (110) and contextual cocaine-seeking behaviors (111), respectively. Overall, this study highlights the potential of these gene alterations in rat whisker follicles as indicators of the reward effects of MA.

We investigated MA-induced transcriptional changes in whisker follicles and the striatum of MA-self-administered rats (47). This study used Molecular Complex Detection (MCODE) cluster analysis on protein–protein interaction (PPI) networks to identify 129 statistically significant core genes [e.g., activity-regulated cytoskeleton-associated protein (ARC), proto-oncogene, and AP-1 transcription factor subunit (Junb)] in whisker follicles and 49 [e.g., amyloid beta precursor protein (App)] in the striatum, potentially serving as diagnostic markers. The DEGs in the striatum were related to nicotine, cocaine, and amphetamine addiction, whereas whisker follicles were associated with Parkinson’s disease, Huntington’s disease, and Alzheimer’s disease. Common genes [period circadian regulator 1 (Per1), DNA damage-inducible transcript 4 (Ddit4), and transgelin (Tagln)] and pathways, including the retrograde endocannabinoid signaling and synaptic vesicle cycle pathways, were identified between the two tissues. This study offers important data on gene expression related to MA reward in whisker follicles and the striatum, potentially aiding research using whisker follicles as alternative biomarkers for diagnosing MA use disorders.

Following research on MA biomarkers in whisker follicles, we extended this study to humans. Gene expression and biomarkers were explored during various withdrawal periods in patients with MUD (49). Transcriptome analysis was performed on hair follicle samples from different MUD stages. Two major clusters were identified: non-recovered (NR) and almost recovered (AR) patients. A predictive model for MUD diagnosis was developed with a high accuracy (98.7% for NR and 81.3% for AR). Important genes such as PMS1 homolog 2, mismatch repair system component (PSM2), and Rac family small GTPase 3 (RAC3) were found. PSM2 was upregulated in NR and linked to neurological dysfunction, while RAC3 downregulation could impact GABAergic neuronal function in disorders such as epilepsy and schizophrenia. This study highlights the potential of transcriptomics-based biomarkers and suggests that previous classification methods for patients with MUDs may have certain limitations. This notable study using hair follicles of patients with MUDs developed a transcriptomic-based predictive model, showing promise for improving MUD diagnosis and advancing future pharmacological treatments for this disorder.

## 5 Conclusion

**Figure 3:**
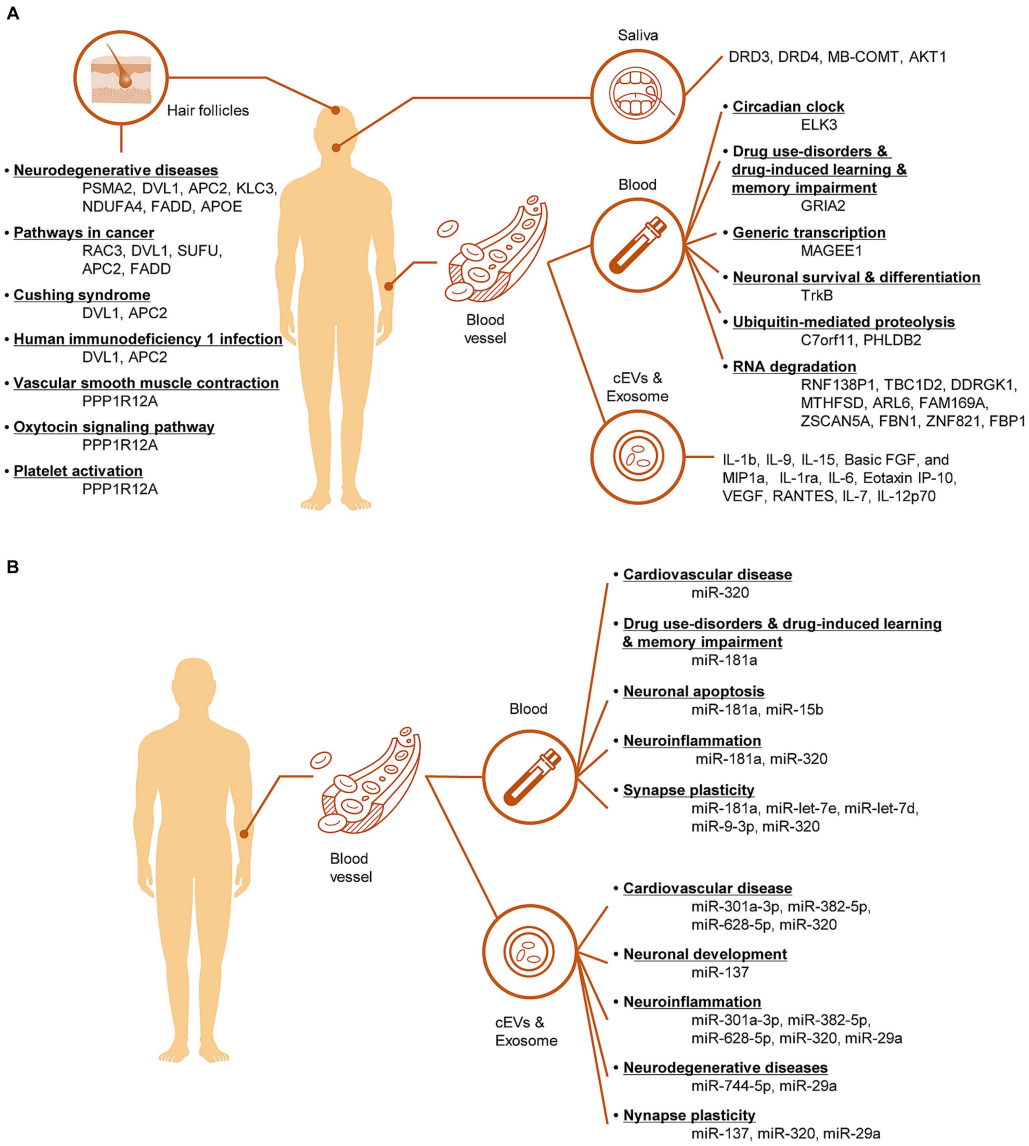
Illustration of the pathological mechanisms involving mRNA biomarkers **(A)** and miRNA biomarkers **(B)** in non-invasive tissues of MUD patients.

This review summarizes the latest advancements in the identification of non-invasive transcriptomic biomarkers for the diagnosis of MUD. Routine MUD diagnosis and discrimination are primarily based on self-reported questionnaires that assess drug use before and during abstinence. However, researchers have expressed concerns about the reliability, validity, and cognitive biases inherent in self-report questionnaires, especially in complex contexts such as SUD. In this respect, we investigated recent trends in the discovery of transcription biomarkers using non-invasive tissues. Non-invasive biomarkers excavated from the blood, exosomes, saliva, hair, and hair follicle cells were investigated, and their functions were described (Figure 3). Moreover, considering gender differences will offer valuable insights into the diagnosis and treatment of MUD. Recently, the development of intrinsic diagnostic markers for MUD in non-invasive tissues has been actively underway in various fields, including transcriptional studies, metabolism, genomics, and proteomics. Multi-omics analyses of these biomarkers, combined with multimodal neuroimaging, can mitigate the diagnostic uncertainties of self-report questionnaires. Moreover, artificial intelligence-powered multi-omics and modal analyses demonstrated high analytical power and reproducibility. Nevertheless, challenges remain for the clinical application of non-invasive biomarkers for the diagnosis of MUD. The difference between laboratory and clinical settings often makes it difficult to directly apply these findings to actual patient care, necessitating verification and standardization to achieve consistent clinical results. Furthermore, integrating MUD transcriptome biomarkers discovered in various studies is challenging due to differences in methodologies, sample types, and experimental conditions used across studies, making it difficult to draw consistent conclusions. Not all reported transcriptome biomarkers are clinically significant or useful. To overcome these issues, the development of more sophisticated analytical tools and the establishment of standardized protocols are necessary. To apply these findings for predicting, diagnosing, and treating MUD, there is a need to explore and agree upon integrated approaches and standards for evaluating clinical utility. Building sustainable diagnostic management pathways and improving the care of MUD patients make non-invasive testing the only viable option for diseases with physical and mental health and socioeconomic implications.

## Data availability statement

The original contributions presented in the study are included in the article/supplementary material, further inquiries can be directed to the corresponding author.

## Author contributions

W-JJ: Data curation, Investigation, Project administration, Visualization, Writing – original draft. SL: Conceptualization, Supervision, Validation, Writing – review & editing. C-HJ: Conceptualization, Funding acquisition, Project administration, Supervision, Writing – review & editing.
